# Preparation of Temperature-Responsive Antibody–Nanoparticles by RAFT-Mediated Grafting from Polymerization

**DOI:** 10.3390/polym14214584

**Published:** 2022-10-28

**Authors:** Erika Yoshihara, Ahmed Nabil, Shinichi Mochizuki, Michihiro Iijima, Mitsuhiro Ebara

**Affiliations:** 1 Research Center for Functional Materials (RCFM), National Institute for Materials Science (NIMS), 1-1 Namiki, Tsukuba 305-0044, Japan; 2 Graduate School of Pure and Applied Sciences, University of Tsukuba, 1-1-1 Tennodai, Tsukuba 305-8577, Japan; 3 Biotechnology and Life Sciences Department, Faculty of Postgraduate Studies for Advanced Sciences (PSAS), Beni-Suef University, Beni-Suef 62511, Egypt; 4 Egyptian Liver Research Institute and Hospital (ELRIAH), El Mansoura 35511, Egypt; 5 Faculty of Environmental Engineering, University of Kitakyushu, 1-1 Hibikino, Wakamatsu, Kitakyushu 808-0135, Japan; 6 Department of Materials Chemistry and Bioengineering, National Institute of Technology, Oyama College (NIT, Oyama College), 771 Nakakuki, Oyama 323-0806, Japan; 7 Graduate School of Industrial Science and Technology, Tokyo University of Science, 1-3 Kagurazaka, Shinjuku, Tokyo 162-0825, Japan

**Keywords:** polymer–protein conjugates, grafting from, temperature-responsive, RAFT, precipitation polymerization

## Abstract

Herein, we report the preparation of temperature-responsive antibody–nanoparticles by the direct polymerization of *N*-isopropylacrylamide (NIPAAm) from immunoglobulin G (IgG). To this end, a chain transfer agent (CTA) was introduced into IgG, followed by the precipitation polymerization of NIPAAm in an aqueous medium via reversible addition–fragmentation chain transfer polymerization above the lower critical solution temperature (LCST). Consequently, antibody–polymer particles with diameters of approximately 100–200 nm were formed. Owing to the entanglement of the grafted polymers via partial chemical crosslinking, the antibody–nanoparticles maintained their stability even at temperatures below the LCST. Further, the dispersed nanoparticles could be collected by thermal precipitation above the LCST. Additionally, the antibody–nanoparticles formulation could maintain its binding constant and exhibited a good resistance against enzymatic treatment. Thus, the proposed antibody–nanoparticles can be useful for maximizing the therapeutic potential of antibody–drug conjugates or efficacies of immunoassays and antibody recovery and recycling.

## 1. Introduction

Protein–polymer conjugates (PPCs) have been applied in a variety of biomedical fields [1]. The introduction of synthetic polymers into biomolecules endows the biomolecules with new functionalities, such as enhanced solubility and dispersibility, immunocompatibility, and inhibition of proteolytic enzymes [2]. Particularly, the modification of proteins with polyethylene glycol (PEG), known as PEGylation, is the first successful technology to improve the pharmacokinetic profiles of therapeutic agents and has been clinically applied for more than 25 years [3]. Other types of synthetic polymers, such as *N*-(2-hydroxypropyl) methacrylamide copolymer [4], poly(vinylpyrrolidone) [5], and poly(2-oxazolines) [6], have also been applied to PPCs. In addition, PPCs have been demonstrated to enable the control of protein–ligand recognition using stimuli-responsive polymers or smart polymers. Stayton et al. demonstrated that the conjugation of a temperature-sensitive polymer to a genetically engineered site on a protein enables the control of the ligand-binding affinity of the protein [7]. This approach can also be applied to light-regulated molecular switches that reversibly control biomolecular function in diagnostics, affinity separations, bioprocessing, therapeutics, and bioelectronics applications [8]. In recent years, the modification of antibodies, such as the synthesis of “antibody–drug conjugates (ADCs)” or “antibody–polymer conjugates (APCs)” has emerged as one of the major challenges of PPCs [9]. For example, the conjugation of PEG to antibodies has demonstrated great effectiveness in prolonging systemic half-life [10]. Additionally, APCs have been applied for the selective delivery of small-interference RNAs to target cells [11]. Furthermore, APC technologies enable the tethering of antibodies to hydrophobic drugs [12,13], contrast agents [14], and phosphors [15]. Antibodies have also been immobilized on the surface of substrates [16,17,18,19] and particles [20]. Particularly, nanoparticulation is considered to improve the payload selection and the amount of drug introduced [21].

There are two major methods of grafting APCs: “grafting to” (GT) and “grafting from” (GF) approaches [22]. The GT approach involves the use of a pre-synthesized polymer to immobilize antibodies via a coupling reaction, whereas the GF approach involves the direct polymerization from an antibody. The GT approach has been widely used in various applications owing to its high coupling reaction efficiency and a wide range of reaction conditions [23,24]. Although *N*-hydroxyester (NHS) chemistry has been commonly used for chemical immobilization, specific interactions, such as streptavidin–biotin-binding have also been used [25,26,27]. More recently, click chemistry has been performed to achieve conjugation under biological conditions [28,29,30]. However, the GT method requires a multi-step synthesis to obtain polymers with functional groups. Additionally, the efficiency of the introduction of polymers into antibodies is limited owing to the occurrence of steric hindrance. To overcome these drawbacks, researchers have recently focused on the GF approach. Several studies have demonstrated the introduction of poly (*N*-isopropyl acrylamide) (PNIPAAm) into bovine serum albumin (BSA) and lysozyme via reversible addition–fragmentation chain transfer (RAFT) polymerization using the GF method [31,32,33].

**Figure 1:**
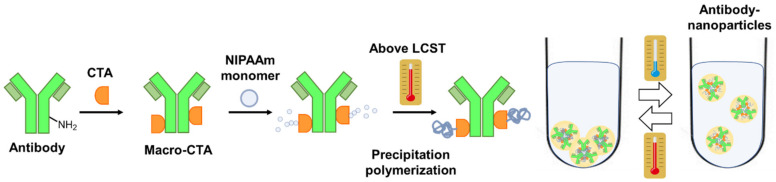
Design concept of the temperature-responsive antibody–nanoparticles prepared using the “grafting from” and “thermal precipitation” methods.

This study aimed to prepare antibody–PNIPAAm conjugates by functionalizing immunoglobulin G (IgG) with chain transfer agent (CTA) groups as an initiation site for the polymerization of NIPAAm. To this end, first, the RAFT agent with the NHS group was reacted with the amino groups of IgG (macro-CTA). Subsequently, NIPAAm monomers were added to initiate direct polymerization from macro-CTA. In this study, polymerization occurred above the lower critical solution temperature (LCST) of PNIPAAm (i.e., thermal precipitation polymerization). This method can enable the synthesis of antibody–polymer conjugates as a uniform particle form (Figure 1). Nanoparticle formation can offer numerous advantages, such as thermal stability, dispersity, and enzymatic resistance. In this study, the introduction of CTA into antibodies and the RAFT polymerization of NIPAAm from the macro-CTA were examined. In addition, the antibody–nanoparticles obtained at different temperatures were characterized. Lastly, the thermal precipitation efficiency and bioactivity of the antibody–nanoparticles were evaluated.

## 2. Materials and Methods

### 2.1. Materials

*N*-Isopropylacrylamide (NIPAAm, Fujifilm Wako Pure Chemical, Japan, 97%) was recrystallized from *n*-hexane and dried under vacuum before use. 2,2′-Azobis [2-(2-imidazolin-2-yl) propane] dihydrochloride (VA-044, Tokyo Kasei, Japan, 98.0%) was recrystallized from methanol and dried under vacuum before use. The following were purchased and used as received: 2-(Dodecylthiocarbonothioylthio)-2-methylpropionic acid *N*-hydroxysuccinimide ester (NHS-CTA, Sigma-Aldrich, St. Louis, MO, USA), *N,N*-dimethylformamide (DMF, 99.5 %, Fujifilm Wako Pure Chemical, Japan), dulbecco’s phosphate buffered saline (PBS, Sigma-Aldrich, St. Louis, MO, USA), goat polyclonal secondary antibody to mouse IgG-H&L (HRP) (IgG, abcam, Japan), dimethyl sulfoxide (DMSO, Fujifilm Wako Pure Chemical, Japan, 99.0%), DL-2-aminobutyric acid (Tokyo Kasei, Japan, 99.0%), fluorescamine (Tokyo Kasei, Japan), sodium ascorbate (Sigma-Aldrich, St. Louis, MO, USA), tris (hydroxymethyl) amino methane (Tris, 99.8%, Sigma-Aldrich, St. Louis, MO, USA), hydrochloric acid (1.0 mol/L, Fujifilm Wako Pure Chemical, Japan), methanol (99.8%, Fujifilm Wako Pure Chemical, Japan), 10×Tris/Glycine/SDS buffer (BIO-RAD, Hercules, CA, USA), coomassie brilliant blue R-250 (CBB, BIO-RAD, Hercules, CA, USA), laemmli sample buffer (BIO-RAD, Hercules, CA, USA), 2-mercaptoethanol (Fujifilm Wako Pure Chemical, Japan, 99%), 2.5 g/L-Trypsin/1 mmol/L-EDTA solution (NACALAI TESQUE, Japan), precision plus protein unstained standards (BIO-RAD, Hercules, CA, USA), goat antibody to mouse IgG (1.96 mg/mL, abcam, Japan), goat antibody to mouse IgG-H&L (2.06 mg/mL, abcam, Japan), mouse IgG (antigen, abcam, Japan), hydrochloric acid (stop solution, goat antibody to mouse IgG (1.96 mg/mL, abcam, Japan), 1.0 mol/L), goat anti-mouse IgG H&L (Biotin) (2 mg/mL, abcam, Japan), streptavidin HRP labeled (1 mg/mL, abcam, Japan), and 3′,5,5′-tetramethylbenzidine (TMB) solution (BIO-RAD, Hercules, CA, USA). Poly (oxyethylene Sorbitan Monolaurate) (Tween 20, Tokyo Kasei, Japan) was used after diluting to a concentration of 0.5% in PBS after purchase. ELISA coating buffer (abcam, Japan) was used after diluting 10 times with ultrapure water after purchase.

### 2.2. Method

#### 2.2.1. Synthesis of IgG-CTA

To enable the conjugation of NHS-CTA and IgG, an alkaline buffer was used to facilitate carbodiimide chemistry. Briefly, the buffer was prepared by dissolving NaHCO_3_ (105 mg, 1.25 mmol) in ultrapure water (resistivity value: 18.2 Ω cm, 20 mL). Thereafter, an aqueous NaOH solution (1 mol L^−1)^ was added dropwise into the buffer until the solution pH reached 8.6. Before conjugation, IgG (400 μg, 0.0026 μmol) was diluted to 1 mg/mL in NaHCO_3_ buffer, after which NHS-CTA (0.26 μmol, 123.1 μg) in DMF (21 μL) was added and the reaction was continued for 24 h in a Block Bath Shaker at 25 °C. After the reaction, the by-products were removed via ultrafiltration (Amicon Ultra Centrifugal Filter, MWCO 10000, 0.5 mL), and the obtained IgG conjugates were washed three times with PBS.

The amount of CTA group introduced was evaluated by measuring the absorbance intensity of the thiocarbonyl group derived at 310 nm using UV–vis spectroscopy, and amino residues were measured using fluorescamine. Briefly, IgG-CTA (0.5 mg/mL, 50 µL) and IgG (0.5 mg/mL, 50 µL) were added to 96-well plates, and fluorescamine dissolved in acetone (50 mg/mL, 5 µL) was added to each well. The fluorescence of each sample was measured using the Infinite M Nano (TECAN, Männedorf, Switzerland) at excitation and measurement wavelengths of 395 and 495 nm, respectively.

#### 2.2.2. Investigation of the Adjustment Conditions of the IgG–PNIPAAm Conjugates

Briefly, the synthesized IgG-CTA (400 μg, 0.0026 μmol) and 200 μL of PBS (pH 7.5) were added to a 1.5-mL microtube. Subsequently, NIPAAm (18 mg, 160 μmol) and 200 μL of PBS containing (3.6 μmol, 1.2 mg) initiator VA-044 were added and dissolved in the previous solution. Thereafter, the reaction was performed in a water bath shaker at 32 °C for 24 h.

#### 2.2.3. Sodium Dodecyl Sulfate Polyacrylamide Gel Electrophoresis (SDS-PAGE)

The obtained conjugates were characterized using SDS-PAGE and compared to IgG and the polymer separately.

#### 2.2.4. Field Flow Fractionation (FFF) Measurement

FFF was performed using an Agilent Technology G131 pumping system (Santa Clara, CA, USA) with an Eclipse 3+ (Wyatt Technology, Santa Barbara, CA, USA), and 150 mM NaCl aq. was used as a mobile phase. The elute was detected using a multiangle light scattering (MALS) detector (DAWN-HELEOS; Wyatt Technology) and a UV detector (G131B; Agilent Technology).

#### 2.2.5. GPC Measurements

Gel permeation chromatography (GPC), and a spectrophotometer. For GPC (Solvent: DMF, Standard: poly (styrene)), the number average molecular weight (Mn), weight average molecular weight (Mw), and molecular weight distribution (Mw/Mn) of the prepared samples were measured.

#### 2.2.6. DLS Measurement

The prepared conjugate solution was diluted and adjusted with PBS to the IgG concentration of 0.05 mg/mL and measured using a Malvern Zetasizer-Nano ZSP at λ = 633 nm, scattering angle 173°, temperature 25 or 35 °C. The diameter and aggregation state of samples were evaluated.

#### 2.2.7. Lower Critical Solution Temperature Measurement

The temperature dependence of the transmittance of the prepared samples was measured using a spectrophotometer. The prepared conjugates solution was diluted and adjusted to 0.0625 mg/mL IgG concentration using PBS. The sample solution and the stirring bar were subjected to absorbance measurement using a spectrophotometer in a nitrogen atmosphere at a wavelength of 450 nm, a temperature range of 25–40 °C, and a temperature increase rate of 0.2 °C/min.

#### 2.2.8. IgG Recovery Ratio Evaluation

The prepared conjugate solution was diluted with PBS to an IgG concentration of 0.5 mg/mL, and 300 μL of the diluted solution was added to a 1.5-mL microcentrifuge tube and centrifuged at 37 °C, 15,000 rpm for 15 min. After aliquoting 240 μL of the supernatant, 240 μL of PBS was added, and the solution was re-dissolved. Subsequently, the IgG concentration of the solution was measured using the BCA method, and the recovery ratio was calculated.

#### 2.2.9. BCA Method

The prepared conjugate solution was diluted with PBS and adjusted to an IgG concentration of 0.05 mg/mL. Subsequently, 25 μL of this solution and 200 μL of the working solution (Micro BCA™ Protein Assay Kit) were added to 96-well plates and incubated at 37 °C for 30 min. The absorbance of each sample was measured at a wavelength of 562 nm, and the BSA concentration was calculated based on the calibration curve of IgG (0, 0.0125, 0.025, 0.05, 0.1 mg/mL).

#### 2.2.10. Enzymatic Degradation Test of IgG–PNIPAAm Using Trypsin

Briefly, trypsin was added to 200 μL of the IgG–PNIPAAm conjugates in PBS (1 mg/mL) and reacted at 25 °C for 24 h. Subsequently, this solution was purified using ultrafiltration (Amicon Ultra Centrifugal Filter, MWCO 10000, 0.5 mL) and measured using FFF.

#### 2.2.11. Evaluation of the Binding Constant of Antibody–Temperature Responsive Polymer Conjugate to Antigen

The apparent binding affinities of IgG–CTA, and IgG–polymer conjugates were measured using a sandwich enzyme-linked immunosorbent assay (ELISA, developed in-house). First, the primary antibody (1.0 mg mL^−1^) was stabilized in a 96-well plate and incubated overnight. Subsequently, the plate was washed five times with PBS containing 0.5% Tween 20. Thereafter, the plate was blocked with 200 μL of blocking buffer and incubated for 30 min, then washed five times with PBS containing 0.5% Tween 20. Next, 100 μL of the antigen (Mouse IgG was used as the model antigen) was added to the 96-well plate by varying the antigen concentration from 10 to 118 pg mL^−1^, after which the plate was incubated for 1 h. Subsequently, the plate was washed five times with PBS containing 0.5% Tween 20. Thereafter, 1 mg mL^−1^ of IgG–CTA biotin-labeled conjugates and IgG–PNIPAAm biotin-labeled conjugates were added and incubated for 30 min, after which HRP-conjugated Streptavidin was added and incubated for 30 min. Subsequently, the binding constants were evaluated. Next, the plate was washed five times with PBS containing 0.5% Tween 20, after which TMB was added. The final assay signals were recorded by measuring the absorbance at 450 nm 10 min after incubation with acid treatment to stop the enzymatic reaction. The binding constants (*K*_B_) were estimated using Scatchard Plot.

$$\left(Kb=\frac{\left[antigen\;captured-antibody\right]}{\left[free\;antibody\right]\left[free\;antigen\right]}\right)$$

## 3. Results and Discussion

### 3.1. Synthesis of IgG-CTA

**Scheme 1:**
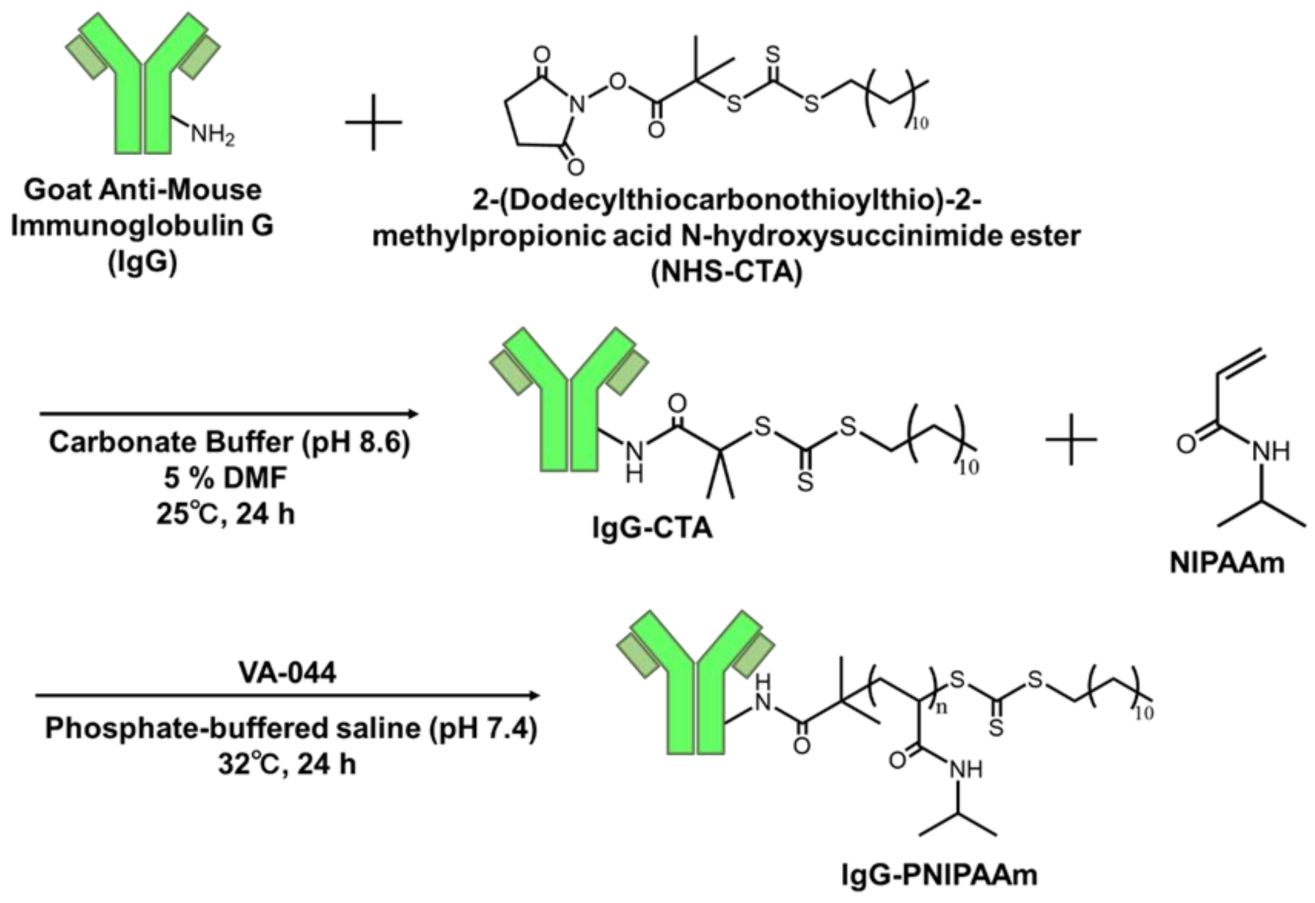
Synthesis of immunoglobulin G–poly (*N*-isopropyl acrylamide) (IgG–PNIPAAm) conjugate by RAFT polymerization.

First, CTA was introduced into IgG (Scheme 1), and the conjugation of the CTA to IgG was confirmed using UV spectroscopy. The absorbance of IgG and IgG-CTA is shown in Figure S1 in the supporting information. This result revealed that IgG-CTA exhibited specific absorption bands at 310 nm, which were derived from thiocarbonyl groups. This is consistent with previous reports regarding the successful introduction of CTAs to IgG [31,33]. Additionally, the successful conjugation was further confirmed by observing the unreacted amino residues in the IgG. The unreacted amino residues were labeled with fluorescamine and their fluorescence intensity was observed (Table S1 in the supporting information). The results revealed that their fluorescence intensity decreased after the introduction of CTA, indicating the successful introduction of CTA groups into the IgG.

### 3.2. Synthesis of IgG–PNIPAAm Conjugates

**Figure 2:**
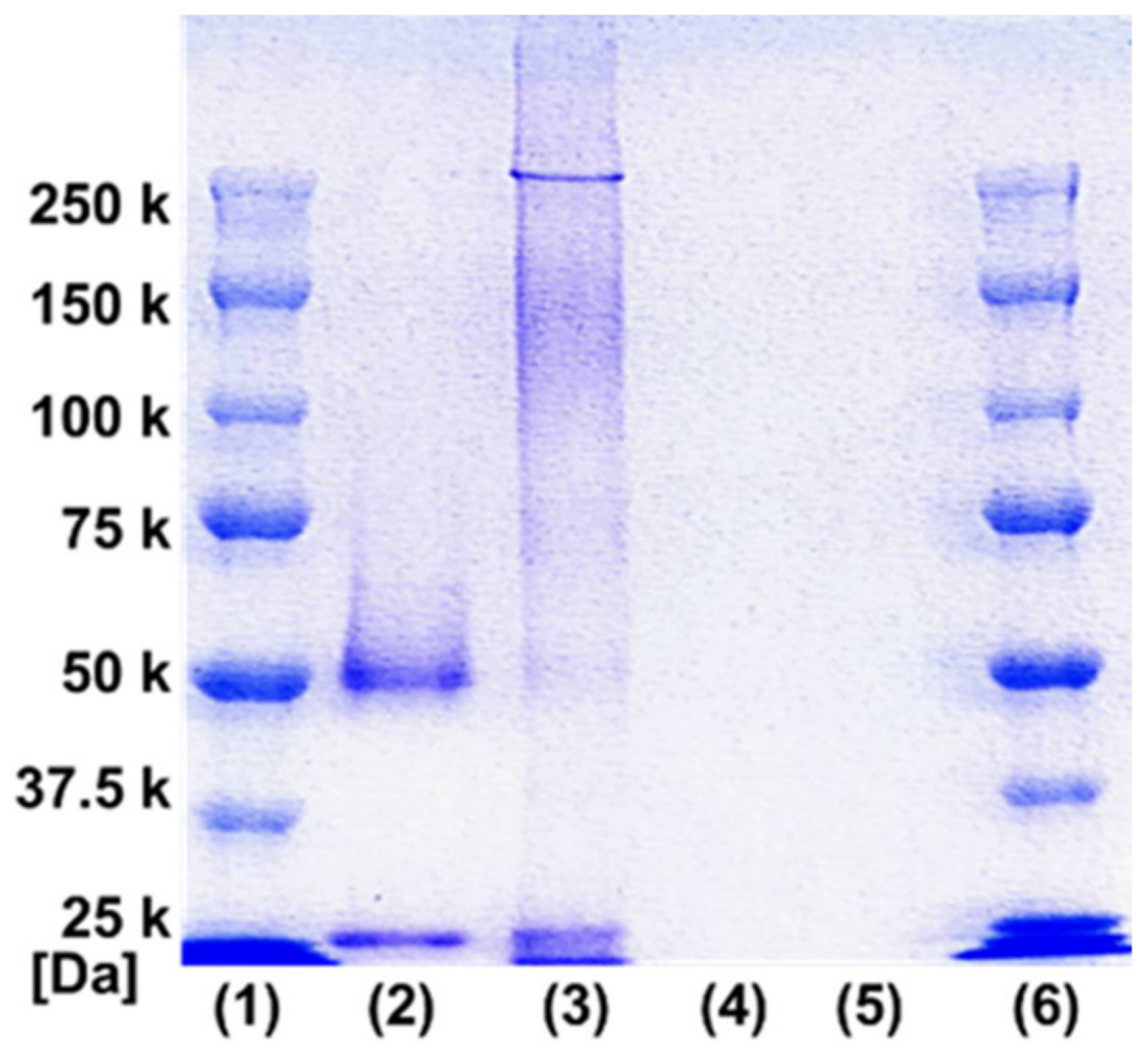
SDS-PAGE analysis results of the IgG–PNIPAAm conjugates: monomer concentration of IgG–PNIPAAm conjugates (lanes: (1) protein standard, (2) IgG, (3) 250 mM, (4) 400 mM, (5) 550 mM, (6) protein standard).

Next, a NIPAAm monomer and an initiator were added to the synthesized macro-CTA (IgG-CTA), and IgG–PNIPAAm conjugates were prepared via RAFT polymerization in PBS. The effects of the monomer concentration (250, 400, and 550 mM) on the physical properties, such as polymerization behavior and thermal precipitation, of the conjugates were investigated. Because the reaction occurred above (or near) the LCST, the precipitates were observed 24 h after the reaction under all conditions. The amount of precipitate formed increased as the monomer concentration increased. To confirm the successful polymerization, SDS-PAGE was performed (Figure 2). The sample containing 250 mM of the monomer exhibited a significant shift of the band to a higher molecular weight than the 50 kDa band of the original IgG fragment. In addition, although the band broadened after the polymerization (100–250 kDa), the band shift was clearly observed, indicating the successful conjugation of PNIPAAm to IgG [29,30]. In contrast, no bands were observed for the samples containing 400 and 550 mM of the monomer because the viscosity of these samples was too high for the electrophoresis. Based on the amino residues, it is estimated that approximately 2–3 mol of CTA is introduced per 1 mol of antibody. Therefore, it was considered that about 2–3 PNIPAAm were introduced into the antibody. Additionally, the reaction of NHS with the amino group of IgG results in Fc-dependent binding has been reported [24] and the SDS-PAGE results confirmed an increase in the molecular weight of only the 5 kDa band which corresponds to the Fc fragment. Hence, the conjugating of polymers to the Fc fragment was expected.

### 3.3. Characterization of IgG–PNIPAAm Using FFF

**Figure 3:**
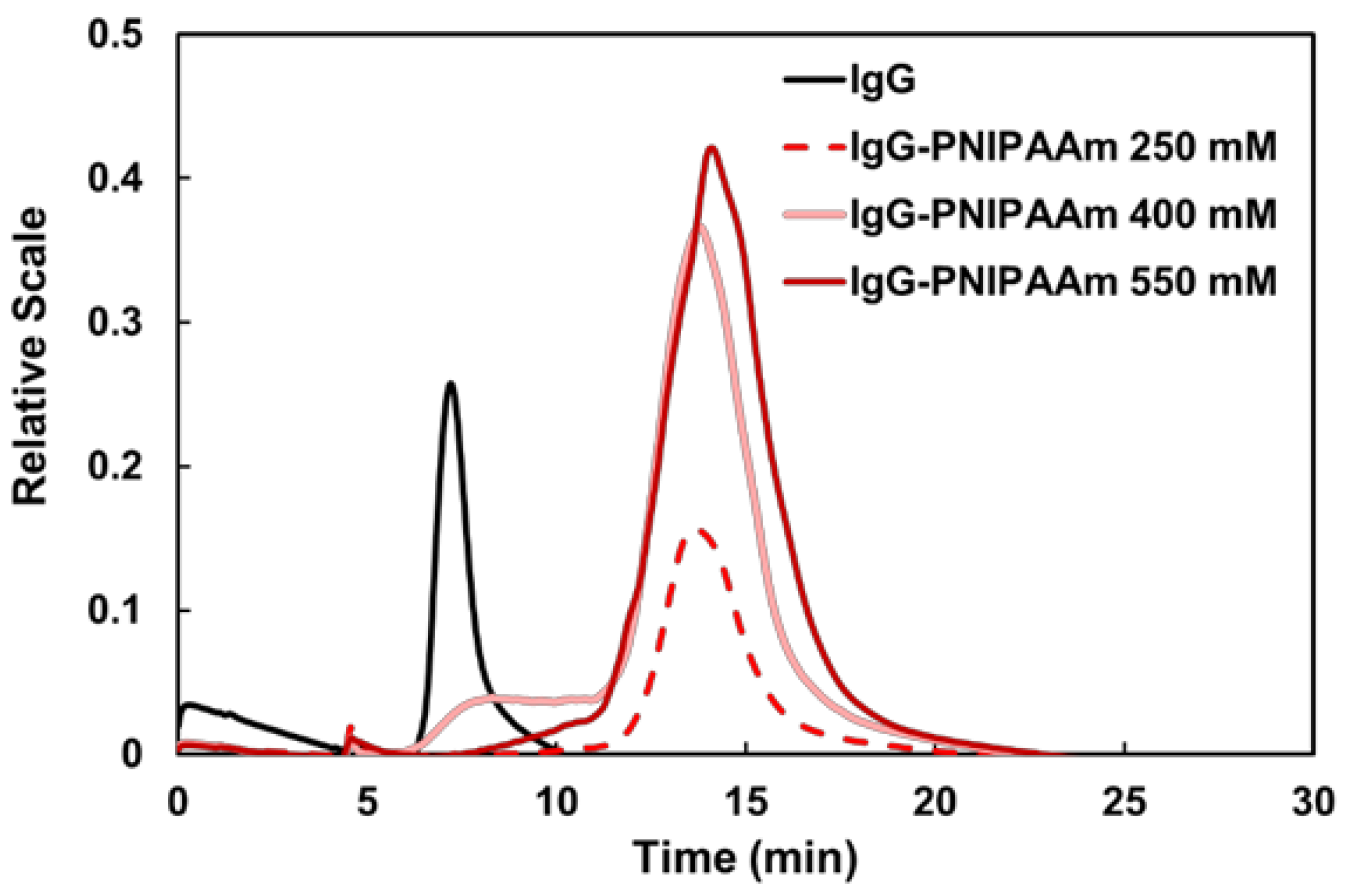
Separation profile of IgG and IgG–PNIPAAm conjugates by FFF.

To confirm the polymerization of NIPAAm from IgG in more detail, the prepared samples were measured using FFF. The FFF technique is a class of analytical techniques that separate analytes based on their hydrodynamic radius owing to the combined action of the laminar flow of a carrier solution, named elution flow, and the flow applied orthogonally to the elution flow, known as cross flow [34]. Figure 3 shows the FFF profiles obtained by measuring the UV absorbance of the samples at 280 nm before and after the conjugation of IgG with PNIPAAm. The elution times of the native IgG and all IgG–PNIPAAm samples were 5–10 and 10–20 min, respectively. As the elution rate increased with a decrease in the particle size, the slow elution time of the polymerized samples indicated an increase in the molecular size of IgG after the conjugation of PNIPAAm. This result demonstrated that the conjugation of PNIPAAm to IgG increased the molecular weight of the samples, indicating that the IgG–PNIPAAm was successfully prepared using the GF approach (Table S2 in the supporting information). Moreover, as shown in Table 1 and Figure 3, the particle size and molecular weight increase with an increase in the monomer concentration. These results suggest that an increase in the monomer concentrations may have resulted in an increase in the molecular weight of the polymer introduced. Furthermore, the SDS-PAGE and FFF results suggest that number of 20–30 IgG-PNIPAAm are gathered together to form nanoparticles.

### 3.4. Evaluation of Antibody—Polymer Conjugates as a Particle Form

**Figure 4:**
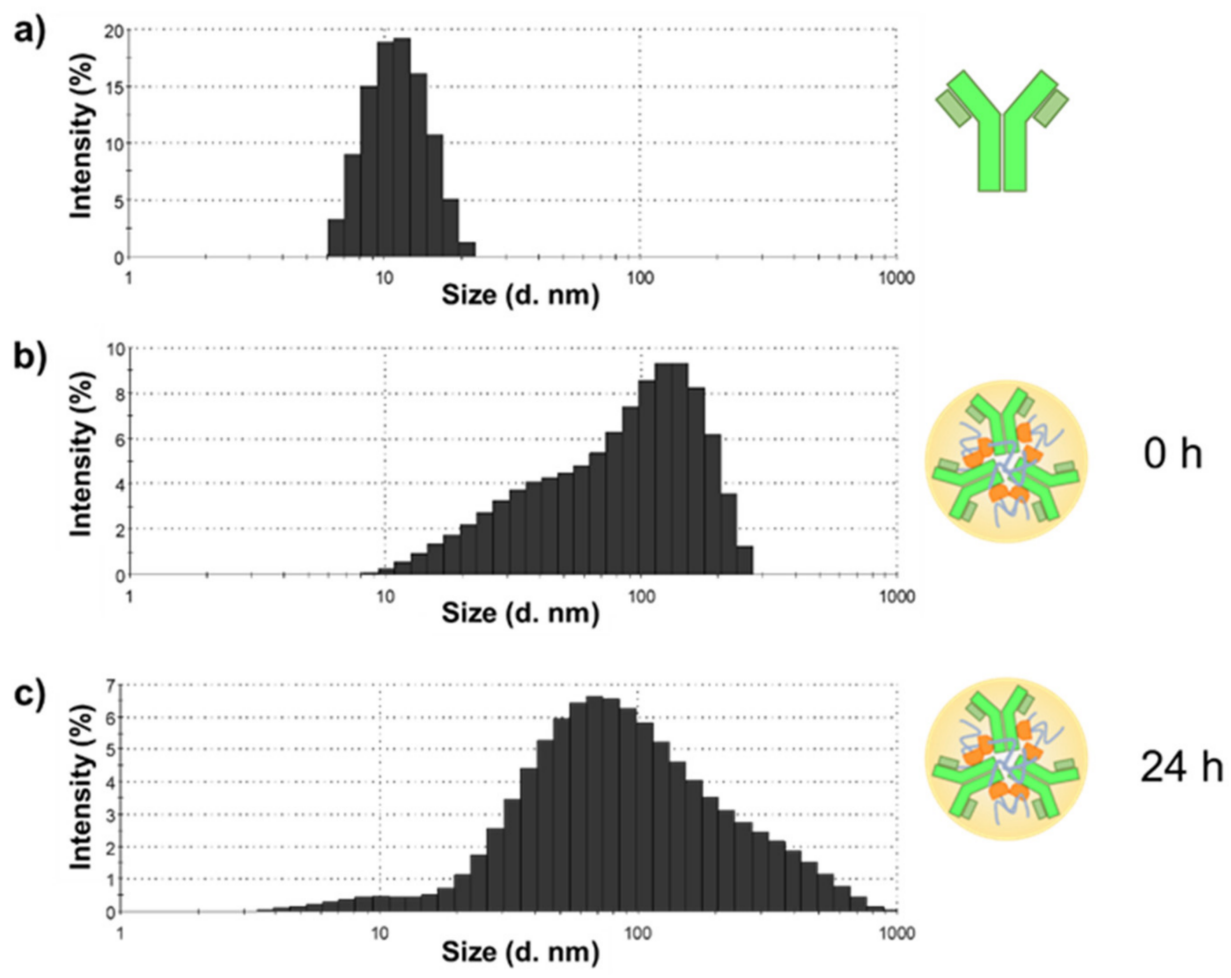
Confirmation of particle size of IgG–PNIPAAm conjugates determined by DLS at 25 °C. (**a**) IgG, (**b**) IgG–PNIPAAm stood for 0 h, (**c**) IgG–PNIPAAm stood for 24 h.

As the particle size of IgG–PNIPAAm was confirmed by FFF, the morphology of IgG–PNIPAAm in water solution was measured by DLS. Figure 4 shows the DLS results of IgG, IgG–PNIPAAm, and IgG–PNIPAAm after standing for 24 h. The particle sizes of IgG and IgG–PNIPAAm were approximately 10 ± 2.3 and 100 ± 53.3 nm, respectively. These results indicate that the conjugation of PNIPAAm to IgG using the GF method resulted in the formation of IgG–PNIPAAm nanoparticles. Moreover, the IgG–PNIPAAm retained its particle form after 24 h of standing, suggesting its high stability (Figure 4b,c). This could be attributed to the hydrophobic interactions of CTA (Figure S3 in the Supporting Information), as well as the entanglement of the grafted polymers. However, several reports have revealed that PNIPAAm chains can undergo “self-crosslinking” by chain-transfer reactions and that microgels can be prepared without the use of a crosslinking agent [35]. The FFF and DLS measurement results indicated the formation and stability of antibody nanoparticles with a size of approximately 100–200 nm. The zeta potential of the antibody was also measured to observe if there was any change in the surface potential of the antibody due to the polymer modification, and no apparent difference was observed (Table S3 in the Supporting Information).

### 3.5. Temperature-Responsive Phase Transition Behavior

**Figure 5:**
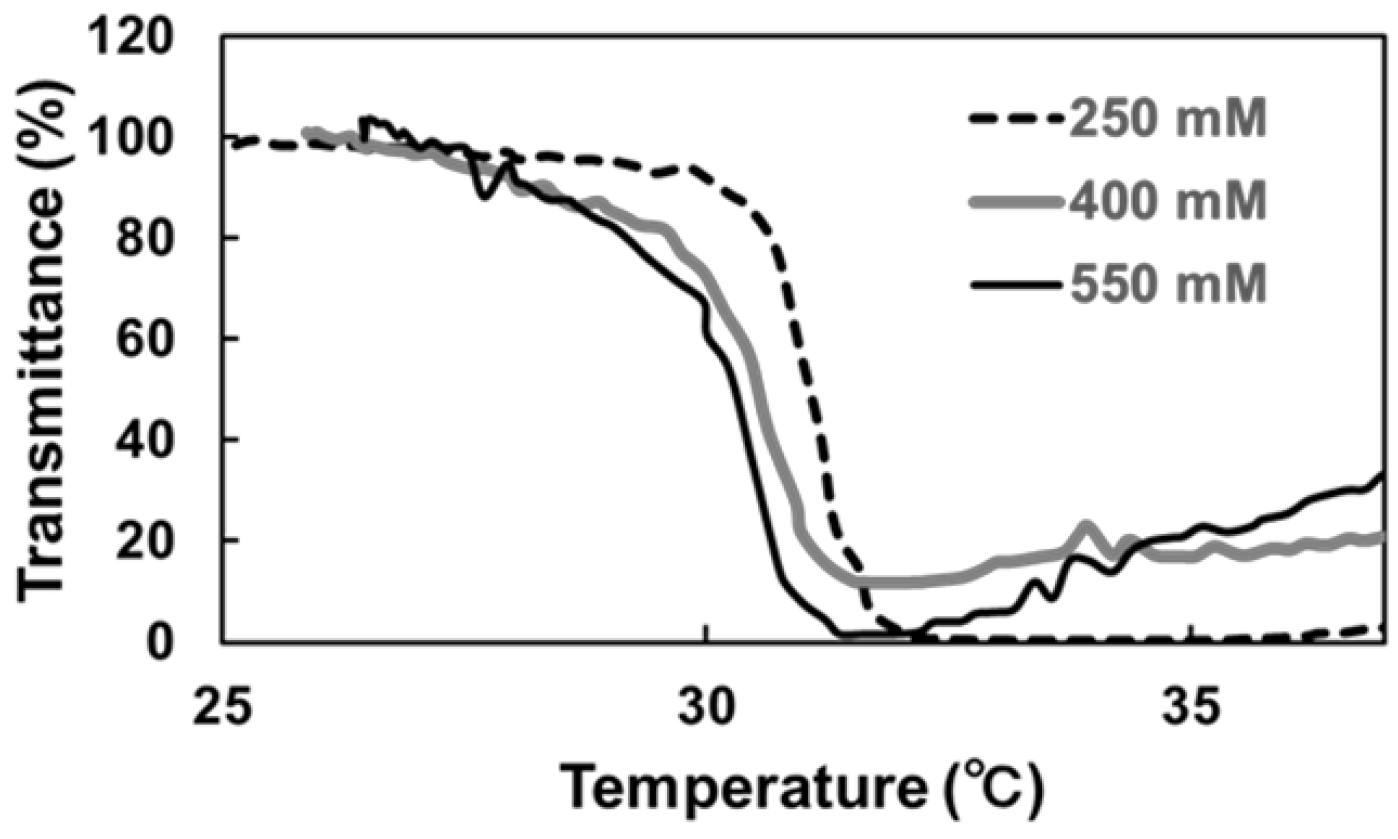
Transmittance of the IgG–PNIPAAm solutions as a function of temperature (thermal-response; solvent: PBS (pH: 7.4), IgG concentration: 0.0625 mg/mL, heating rate: 0.2 °C/min, wavelength: 450 nm).

The temperature-responsive phase transition of the conjugates before and after the polymeric conjugation was observed by measuring the LCSTs. LCST was defined as the temperature at which 50% transmittance was observed. The LCST of the conjugate was observed at approximately 31.0 °C, confirming the temperature responsivity of the prepared conjugates (Figure 5). Generally, LCST tends to shift to a higher temperature when PNIPAAm is conjugated to proteins using the GT method because unreacted functional groups may contribute to increase the hydrophilicity. In our previous studies, the LCST of antibody–PNIPAAm conjugates prepared using the GT method was 37.0 °C [29,30]. On the other hand, the LCST of a BSA–PNIPAAm conjugate prepared using the GF method has been reported to be 31.0 °C, which is consistent with the findings of this study [33]. This indicates that the polymerization of homo NIPAAm from proteins without the presence of any co-monomers may be one of the advantages of the GF method. Additionally, the unconjugated free PNIPAAm in the solution can contribute to maintain the sharp phase transition of the conjugates.

### 3.6. Response Evaluation of IgG–PNIPAAm Nanoparticles

**Figure 6:**
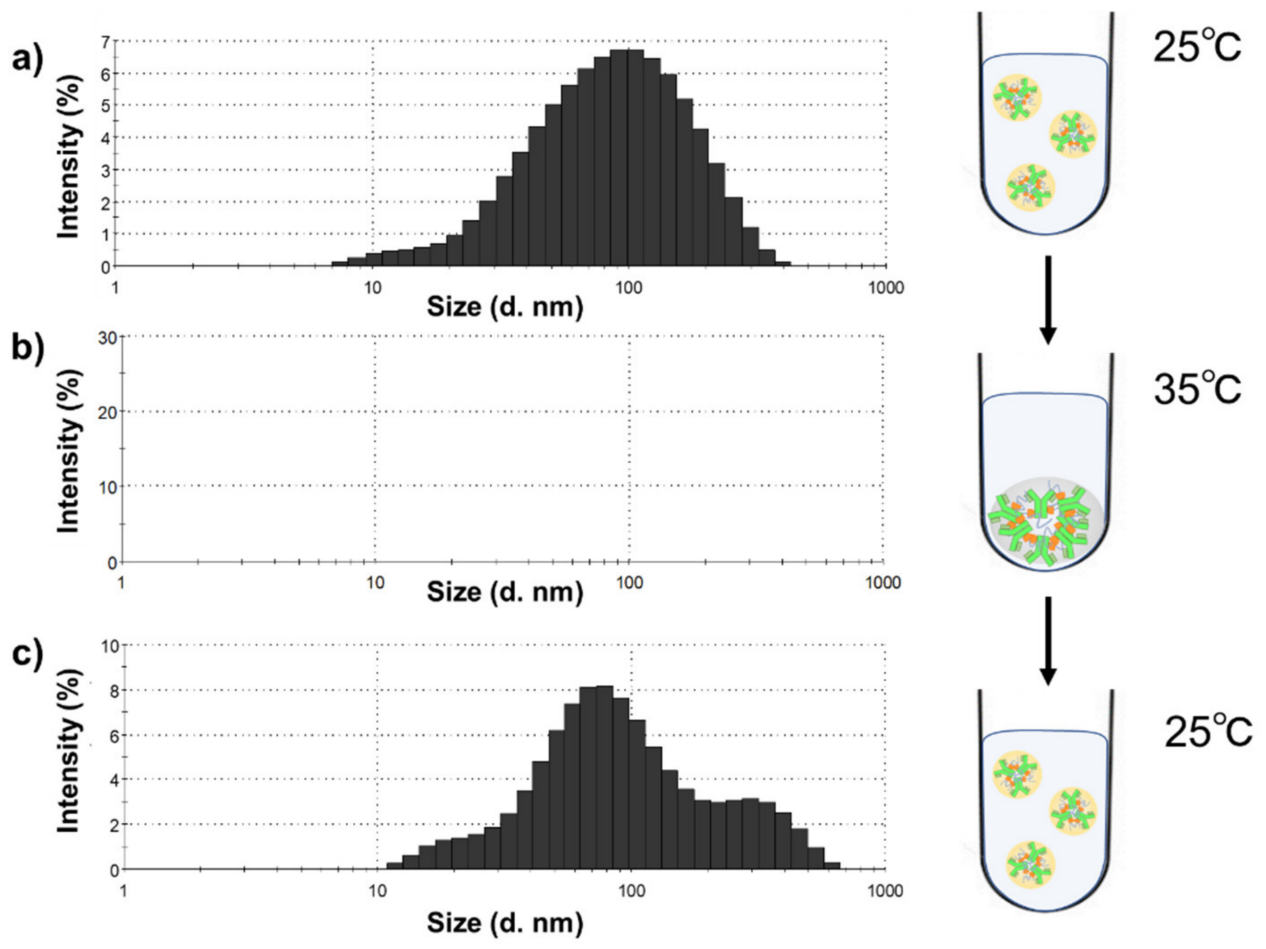
Confirmation of the particle size of IgG–PNIPAAm conjugates in response to temperature using DLS. (**a**) 25 °C, (**b**) 35 °C, (**c**) 25 °C.

To confirm the reversibility of particle formation in response to temperature, the IgG–PNIPAAm conjugates were measured above the LCST using DLS. First, the sample was measured at 25 °C, after which the temperature was increased to 35 °C (above LCST), and the sample was measured. Lastly, the temperature was returned to 25 °C, and the sample was measured (Figure 6). From Figure 6b, the spectrum of IgG–PNIPAAm disappeared at 35 °C. This is because the particles aggregated and precipitated from the solution. In contrast, with a decrease in the temperature to 25 °C, the particle formation was confirmed again. The results revealed that the particles of IgG–PNIPAAm conjugates exhibited an “on–off” switch functional ability with a change in temperature.

### 3.7. Thermal Precipitation and Recovery of IgG–PNIPAAm

**Figure 7:**
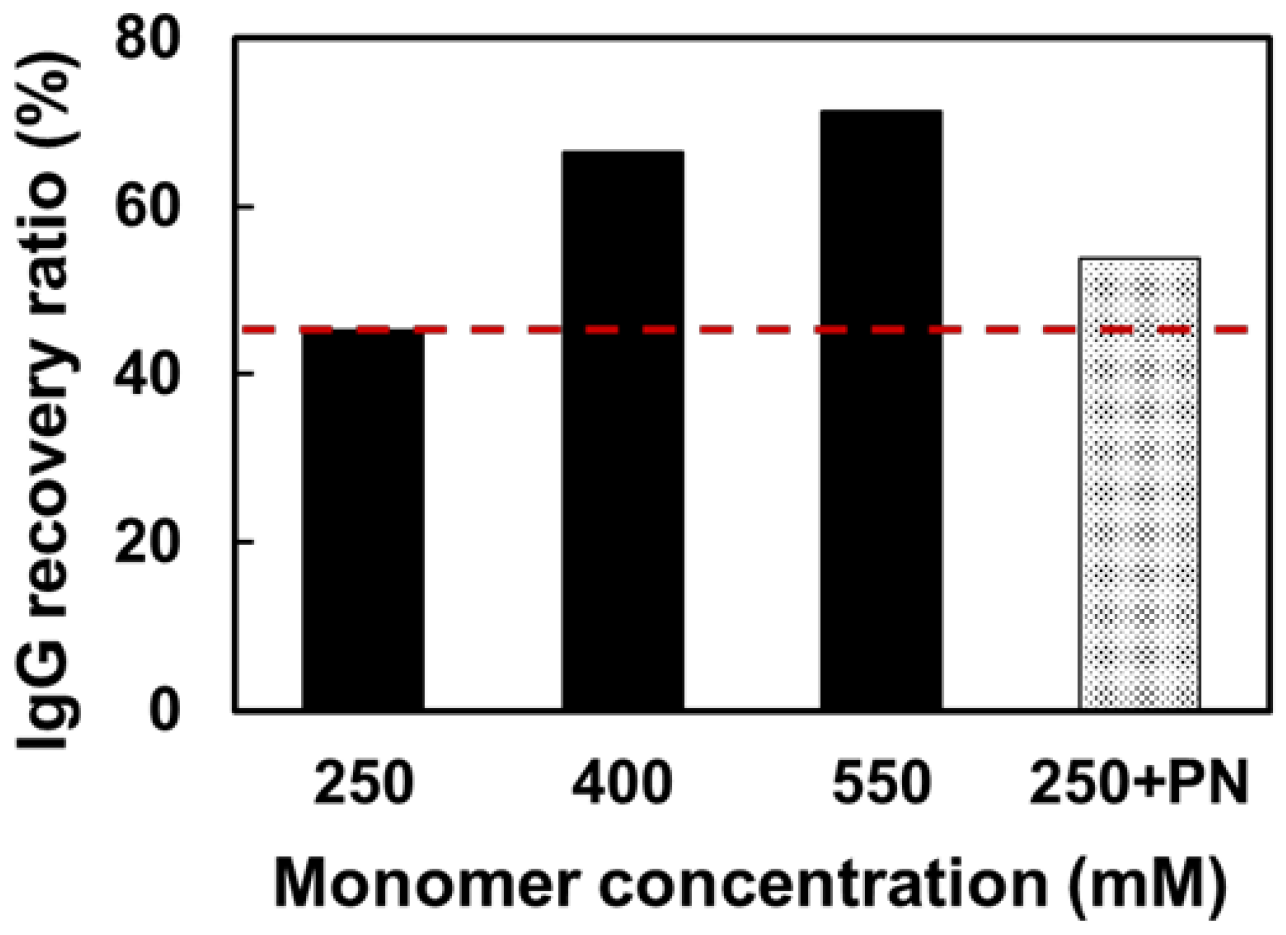
Effects of monomer concentration on IgG recovery ratio (PN: free PNIPAAm).

To evaluate whether the monomer concentration of IgG–PNIPAAm affects the thermal precipitation efficiency, the recovery ratio of IgG was measured. Figure S4 shows the thermal precipitation protocol of IgG–PNIPAAm. The recovery ratio was calculated from the balance between the original solution and the supernatant solution. The IgG recovery at monomer concentrations of 250, 400, and 550 mM were 45, 67, and 71%, respectively (Figure 7). The results suggested that the thermal precipitation efficiency increased with increasing monomer concentration. However, under high monomer concentrations, the time taken to re-dissolve after precipitation increased. Therefore, we also examined another approach using free-PNIPAAm to achieve highly efficient thermal precipitation at a low monomer concentration. PNIPAAm homopolymer was added to the solution to enhance the co-sedimentation effect. When PNIPAAm was added to a sample containing 250 mM of the monomer, IgG recovery was improved by approximately 10%. These data suggest that the net amount of free PNIPAAm in solution affects the thermal precipitation efficiency. The obtained recovery ratio was higher than those in previously reported systems that utilized the GT method [29,30]. This could be attributed to the high efficiency of the introduction of macromolecules into the antibody via the GF method compared to the GT method, resulting in higher thermal precipitation efficiency.

### 3.8. Evaluation of the Stability of the Nanoparticles against Enzymes

**Figure 8:**
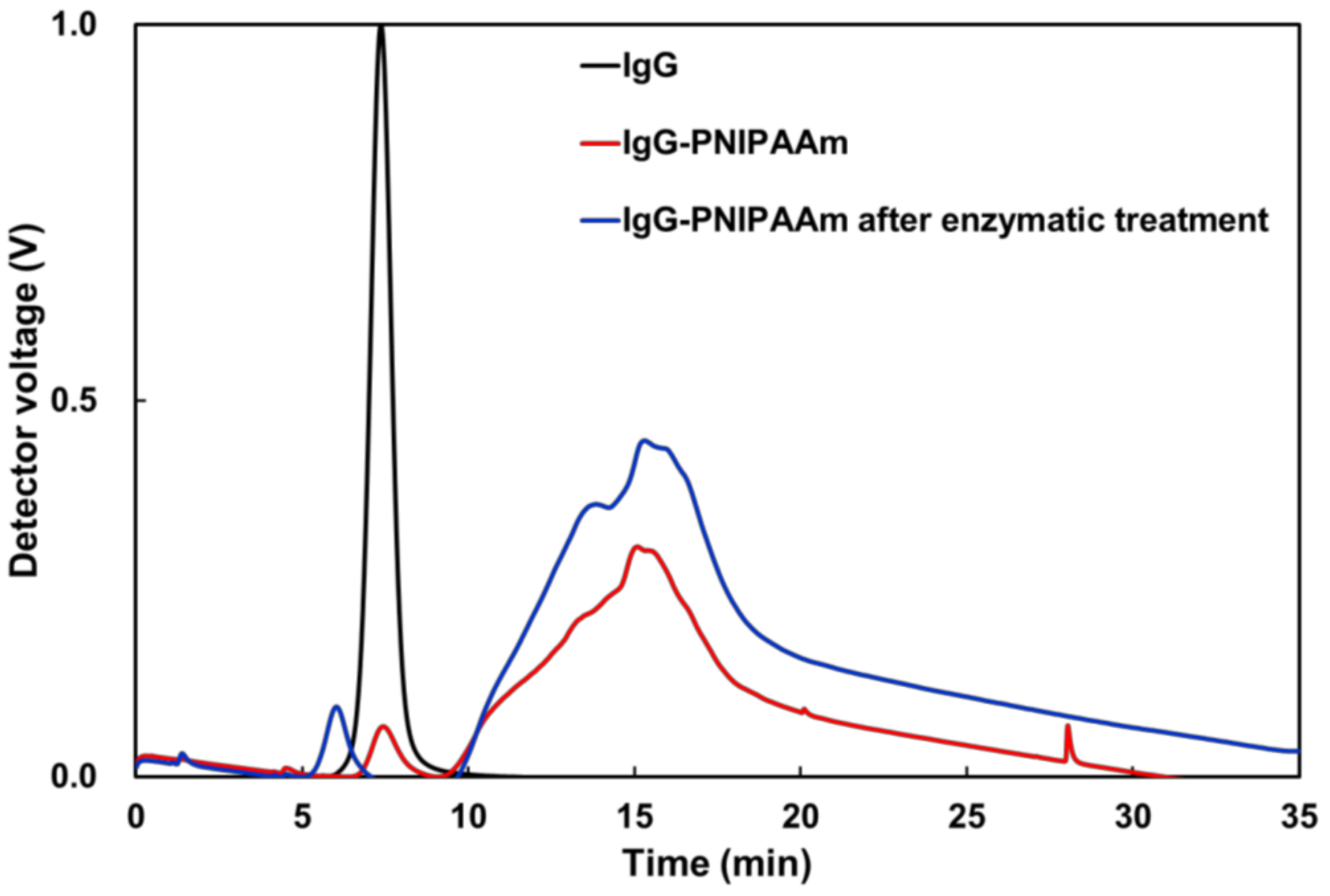
Separation profile of IgG and IgG–PNIPAAm conjugates before and after enzymatic treatment by FFF.

One of the advantages of particle formation is that it can protect antibodies from enzymatic degradation. Here, to evaluate the degradation stability of the nanoparticles against enzymes in the body, the enzymatic degradation of the prepared IgG–PNIPAAm conjugates in the presence of trypsin was confirmed under limited acceleration test conditions. The IgG–PNIPAAm conjugates before and after treatment with the enzyme were measured using FFF (Figure 8). A small peak, which overlaps with a peak of native IgG, was observed in the IgG–PNIPAAm chromatogram at approximately 7–8 min, indicating that the IgG–PNIPAAm contains some unreacted IgG molecules. The spectrum of IgG–PNIPAAm after enzymatic treatment revealed that the elution time of the unreacted IgG’s peak (elution time; 6 min) decreased, indicating the degradation of the unmodified IgG by trypsin under this experiment conditions. However, there was no shift in the peak of the IgG–PNIPAAm conjugate (elution time; 10–20 min) before and after the enzyme treatment, suggesting that the particle form can protect antibodies from enzymatic degradation. These data indicated that this antibody–nanoparticle inhibits the action of trypsin.

### 3.9. Evaluation of the Binding Constant of the Antibody-Temperature Responsive Polymer Conjugate to Antigen

**Figure 9:**
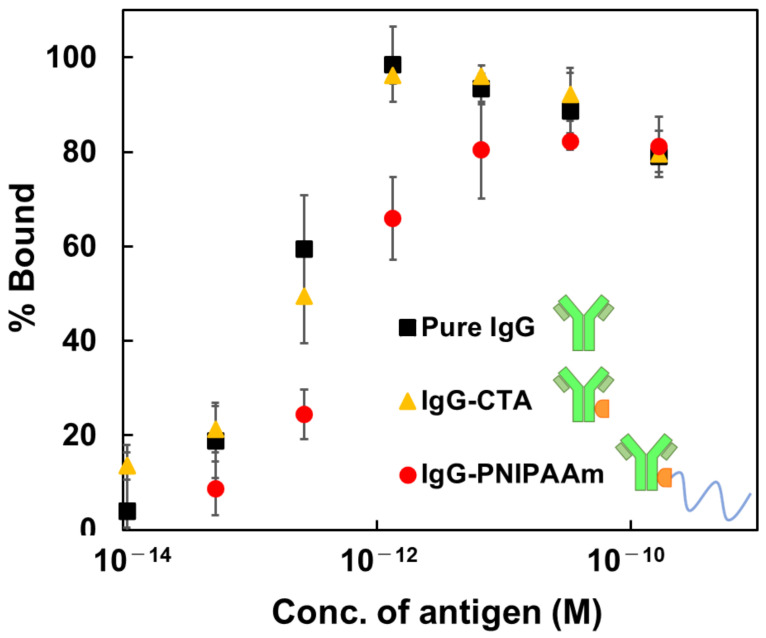
Effect of antigen concentration on the antigen–antibody reaction measured using ELISA (mean ± SD, n = 3).

Lastly, the bioavailability of the conjugated antibody was evaluated because one of the concerns of the APC technique is that introduction of polymers into antibody may decrease the binding constant. The apparent binding affinities of IgG–CTA and IgG–polymer conjugates were assessed using a sandwich ELISA (developed in-house). The ELISA result of the free antigen is summarized in Figure 9. The binding constants were estimated using the Scatchard Plot, as described in the Section 2. Generally, the binding constant, *K*_B_, of an antibody is known to be 10^12^ L mol^−1^. In this study, the estimated binding constants of IgG–CTA and IgG–polymer conjugates were *K*_B_ = 4.19 × 10^12^ L mol^−1^ and *K*_B_= 2.00 × 10^12^ L mol^−1^, respectively. Although the maximum binding ratio of IgG–PNIPAAm was slightly reduced to approximately 80%, it is presumed that the activity of the antigen–antibody reaction was sufficiently retained. These results indicate that the introduction of polymers using the GF method exerts a slight effect on the activity of antibodies. This investigation suggests the superiority of the GF method, as the introduction of polymers using the GT method in the previous study significantly reduced the binding ratio (*K*_B_ = 1.40 ± 0.17 × 10^7^ L mol^−1^) [29]. This explains the formation of nanoparticles by the IgG–PNIPAAm conjugates. It was reported that immobilizing antibodies on the surface of nanoparticles can enhance the signal of ELISA [36]. This suggests that the sensitivity of the ELISA was improved in this study compared to the previous study. To increase the sensitivity of ELISA, protocols generally require several steps to immobilize antibodies on the surface of gold or silica nanoparticles [36,37]. However, in this research, antibody particles can be produced in a single step, making this system highly versatile.

Furthermore, the stability evaluation of the enzyme described previously suggests that PNIPAAm chains may inhibit the enzyme, but this section indicates that the antigen–antibody reaction is not affected by the introduction of PNIPAAm. This could be attributed to the possible conjugation of the polymer to the Fc fragment of the antibody. In conclusion, this material is expected to be applied in various systems such as APCs, immunoassays, recycling of antibodies, etc.

## 4. Conclusions

This study successfully synthesized IgG–PNIPAAm conjugates using the GF strategy. First, analysis results revealed that the properties of IgG–PNIPAAm conjugates differed with a change in the monomer concentration: a high thermal precipitation efficiency was observed at a high monomer concentration. In addition, the IgG–PNIPAAm conjugates exhibited stability against enzymes. Lastly, the immunoaffinity of the IgG–PNIPAAm was evaluated, and the results revealed that the strategy proposed in this study suppressed the decrease in the binding constant associated with polymer conjugation. To the best of our knowledge, this research is the first to prepare IgG–PNIPAAm via RAFT polymerization using the GF method. The proposed technology is efficient for introducing polymers into proteins, providing a wider range of applications in the medical field than traditional methods. In addition, this system can be used for APCs, bioseparation, and diagnostic approaches.

## Figures and Tables

**Table 1:** Characterization of IgG–PNIPAAm by field flow fractionation (FFF).

Monomer Concentration in Feed (mM)	Molecular Weight (g/mol)	Radius of Inertia (nm)
250	9.9 × 10⁶	104.2
400	1.3 × 10⁷	129.9
550	1.4 × 10⁷	132.5

## Data Availability

Not applicable.

## Supplementary Materials

The following supporting information can be downloaded at: https://www.mdpi.com/article/10.3390/polym14214584/s1, Table S1: Value of each measurement by UV-vis and Fluorescence intensity; Figure S1: UV-vis measurement results of IgG and IgG-CTA in PBS (0.1 mg/mL); Figure S2: The GPC results of the model reaction between CTA and monomer; Table S2: The GPC results of each model reaction between NHS-CTA and monomer; Figure S3: DLS measurement result of IgG-CTA in PBS (0.1 mg/mL); Table S3: Value of each measurement by DLS and Zeta Potential; Figure S4: The thermal precipitation protocol of IgG-PNIPAAm.
