# Supplementary material for: Preparation of Temperature-Responsive Antibody–Nanoparticles by RAFT-Mediated Grafting from Polymerization
Source: Polymers (Basel). 2022 Oct 28;14(21):4584. doi: 10.3390/polym14214584 (PMC9657504; doi:10.3390/polym14214584)
Supplement: Supplementary file 1 [file polymers-14-04584-s001.zip › polymers-1980588-supplementary.pdf]

Supporting Information

Preparation of Temperature-responsive Antibody-nanoparticles by RAFT mediated Grafting from Polymerization

Erika Yoshihara [1,2], Ahmed Nabil [1,3,4], Shinichi Mochizuki [5], Michihiro Iijima [6] and Mitsuhiro Ebara [1,2,7] *

[1] Research Center for Functional Materials (RCFM), National Institute for Materials Science (NIMS), 1-1 Namiki, Tsukuba 305-0044, Japan

[2]Graduate School of Pure and Applied Sciences, University of Tsukuba, 1-1-1 Tennodai, Tsukuba 305-8577, Japan

[3]Biotechnology and Life Sciences Department, Faculty of Postgraduate Studies for Advanced Sciences (PSAS), Beni-Suef University, Beni-Suef 62511, Egypt

[4]Egyptian Liver Research Institute and Hospital (ELRIAH), El Mansoura, 35511, Egypt

[5]The University of Kitakyushu Faculty of Environmental Engineering, 1-1 Hibikino, Wakamatsu, Kitakyushu, Japan

[6]Department of Materials Chemistry and Bioengineering, National Institute of Technology, Oyama College (NIT, Oyama College), 771 Nakakuki, Oyama 323-0806, Japan

[7]Graduate School of Industrial Science and Technology, Tokyo University of Science, 1-3 Kagurazaka, Shinjuku, Tokyo 162-0825, Japan

*** E-mail: EBARA.Mitsuhiro@nims.go.jp**

Table of contents:

Table S1. Value of each measurement by UV-vis and Fluorescence intensity.

Figure S1. UV-vis measurement results of IgG and IgG-CTA in PBS (0.1 mg/mL).

Figure S2. The GPC results of the model reaction between CTA and monomer**.**

Table S2. The GPC results of each model reaction between NHS-CTA and monomer.

Figure S3. DLS measurement result of IgG-CTA in PBS (0.1 mg/mL).

Table S3. Value of each measurement by DLS and Zeta Potential.

Figure S4. The thermal precipitation protocol of IgG-PNIPAAm.

Table S1. Value of each measurement by UV-vis and Fluorescence intensity.

	Abs. (310 nm/280 nm) *	Fluorescence intensity (495 nm) **
IgG	0.018	26130
IgG-CTA	0.080	25047

* The absorbance intensity derived from thiocarbonyl group (310 nm) against absorbance derived from protein (280 nm).

** The fluorescence intensity derived from fluorescamine (495 nm) conjugated to unreacted amino residues.

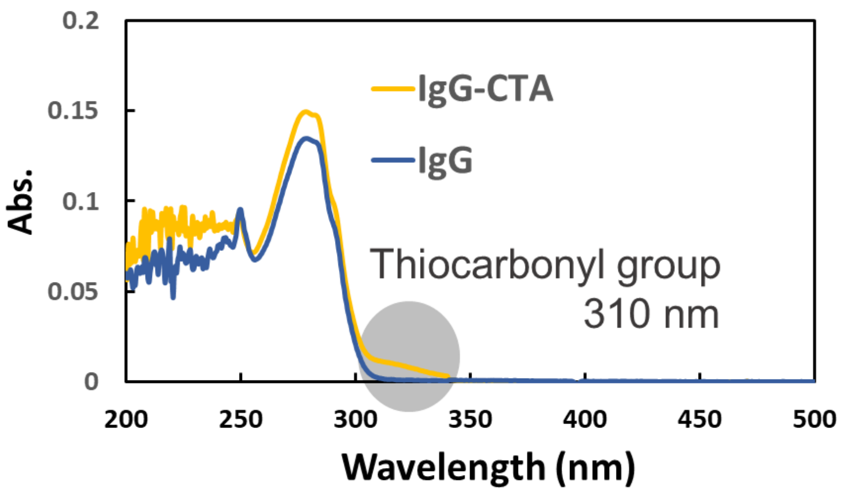

Figure S1. UV-vis measurement results of IgG and IgG-CTA in PBS (0.1 mg/mL).

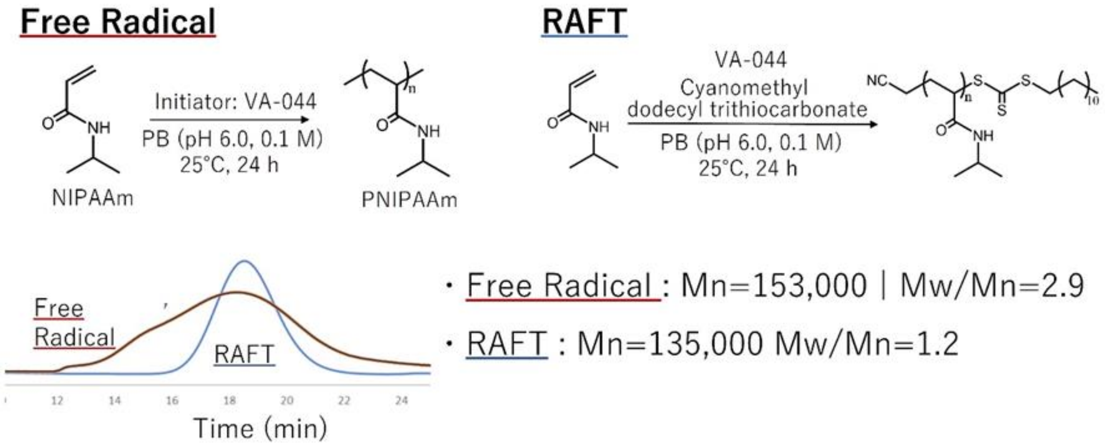

Figure S2. The GPC results of the model reaction between CTA and monomer.

Table S2. The GPC results of each model reaction between NHS-CTA and monomer.

Monomer concentration (mM)	Mn	Mw/Mn
250	1023,000	1.8
400	1118,000	1.8
550	1474,000	1.5

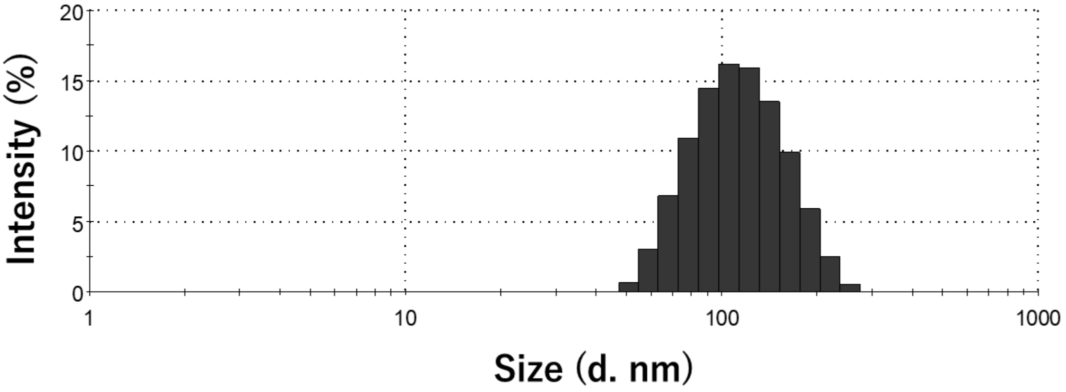

Figure S3. DLS measurement result of IgG-CTA in PBS (0.1 mg/mL).

Table S3. Value of each measurement by DLS and Zeta Potential.

Sample name	Size (d.nm)	Zeta Potential (mV)
IgG	10 ± 2.3	- 6.0
IgG-CTA	118 ± 19.5	- 1.3
IgG-PNIPAAm	100 ± 53.3	- 1.1

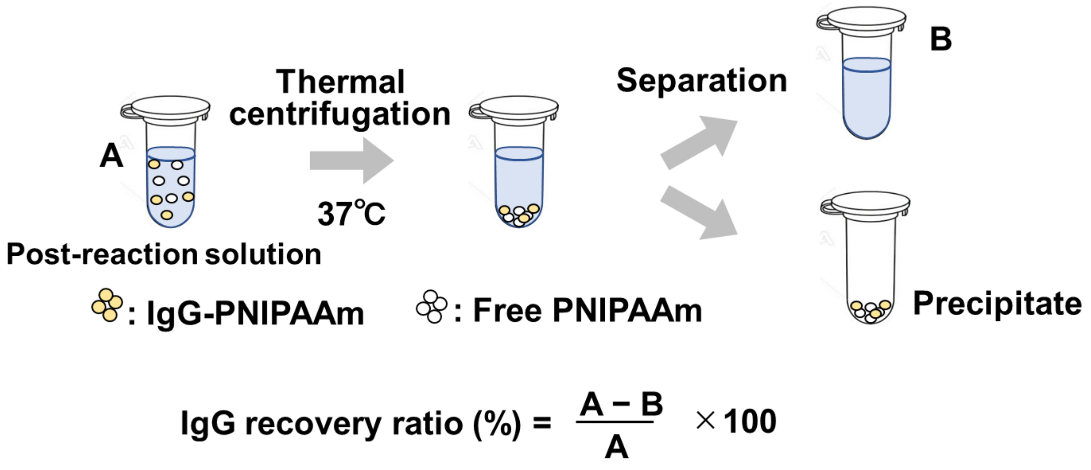

Figure S4. The thermal precipitation protocol of IgG-PNIPAAm.
